# Pneumonitis and pulmonary fibrosis in a patient receiving adjuvant docetaxel and cyclophosphamide for stage 3 breast cancer: a case report and literature review

**DOI:** 10.1186/1752-1947-6-413

**Published:** 2012-11-30

**Authors:** Roberto Ochoa, Pablo A Bejarano, Stefan Glück, Alberto J Montero

**Affiliations:** 1 Sylvester Comprehensive Cancer Center, University of Miami, Leonard M. Miller School of Medicine, 1475 N.W. 12th Avenue Suite 3410, Miami, FL 33136, USA; 2 Department of Pathology, University of Miami/JMH, 2042D JMH-Holtz Ctr, Miami, FL 33136, USA

## Abstract

**Introduction:**

Pulmonary toxicities associated with chemotherapeutic agents utilized as adjuvant therapy in patients with breast cancer are distinctly uncommon. The chemotherapy regimen of docetaxel/cyclophosphamide has a more favorable therapeutic index compared to anthracycline-based regimens due to a significantly lower incidence of heart failure and leukemia. Consequently, docetaxel/cyclophosphamide is the preferred adjuvant chemotherapy of choice in older women or in women where anthracyclines may be contraindicated. Pulmonary complications in patients with breast cancer receiving taxane-based adjuvant chemotherapy in the absence of radiation are distinctly uncommon. Here, we report the case of a patient receiving adjuvant docetaxel/cyclophosphamide who developed rapid-onset, biopsy-proven interstitial pneumonitis.

**Case presentation:**

A 72-year-old Hispanic woman was diagnosed as having stage 3 hormone-receptor positive, human epidermal growth factor receptor 2/neu negative, invasive breast cancer. Due to the estimated 10-year risk of recurrence of approximately 80 percent, a decision was made to treat our patient with adjuvant chemotherapy. Due to her age and increased risk of cardiac toxicity with anthracycline-based chemotherapy regimens, our patient was treated with docetaxel/cyclophosphamide chemotherapy for a total of four planned cycles. However, approximately two weeks after receiving the third cycle of chemotherapy, our patient developed rapidly progressive dyspnea, and a non-productive cough and went to the emergency room at an outside medical facility. She was found to have mild hypoxemia, and new onset of peripheral, subpleural fibrotic changes not present on pre-treatment scans. A thorascopic-guided wedge biopsy of the lung tissue revealed subacute interstitial pneumonitis. Our patient made a rapid clinical recovery after treatment with corticosteroids.

**Conclusions:**

Interstitial pneumonitis is a rare complication of docetaxel/cyclophosphamide chemotherapy that carries a high mortality rate. The only way to make a definitive diagnosis is with a wedge biopsy of the lung, which should be performed when feasible. Our patient’s case illustrates that no therapeutic intervention is without its intrinsic and unanticipated risks, and interstitial pneumonitis should be discussed as a potential side effect with all patients prior to administering docetaxel/cyclophosphamide chemotherapy.

## Introduction

One of the primary disadvantages of most cytotoxic chemotherapeutic agents is their narrow therapeutic index due to off target adverse effects [1]. While the most common chemotherapy-related toxicities are typically self-limiting, others are much less common, and potentially life threatening. One of the most feared toxicities of adjuvant chemotherapy in patients with breast cancer is anthracycline-induced cardiac toxicity, and therapy-induced leukemia. Lung-related toxicities with chemotherapeutic agents used in breast cancer are distinctly uncommon, however they are well known with drugs used in other solid tumors, for example, bleomycin and methotrexate [2].

Docetaxel/cyclophosphamide (TC) has been shown in a prospective randomized trial to be superior to doxorubicin/cyclophosphamide (AC) in improving overall and disease-free survival in patients with breast cancer, with a more favorable therapeutic index due to a significantly lower incidence of heart failure and leukemia [3]. TC is consequently the preferred adjuvant chemotherapy of choice in women with lymph-node negative breast cancer, and when anthracyclines are contraindicated. Pulmonary complications are not commonly associated with docetaxel or cyclophosphamide, and only a small number of cases have been reported in the literature [4-21]. Here, we present the case of a patient receiving adjuvant TC who developed rapid-onset interstitial pneumonitis, illustrating that no therapeutic intervention is without its intrinsic and unanticipated risks.

## Case presentation

A 72-year-old non-smoking Hispanic woman was initially diagnosed as having a large right breast mass measuring 11×7×11cm on physical examination, and axillary adenopathy. A core biopsy of the right breast and axillary lymph nodes revealed infiltrating lobular breast carcinoma with metastasis to the axilla. The tumor was both estrogen-receptor and progesterone-receptor positive (ER+/PR+) with Allred scores of 8 and 3, respectively. Immunostains for human epidermal growth factor receptor 2 (HER2)/neu were negative. Aside from the breast and axillary masses, the remainder of her physical exam was unremarkable. A subsequent staging bone scan and computed tomography scan did not demonstrate any evidence of distant metastases, and she was therefore clinically staged as T3, N2 M0. She was given pre-operative endocrine therapy with exemestane (25mg) daily. Our patient tolerated exemestane well with a clinical reduction in the size of the breast mass. After approximately six months after starting exemestane, our patient underwent a right modified radical mastectomy and axillary lymph node dissection. Histological examination revealed extensive tumor (ypT3ypN3) with 17 out of 20 lymph nodes involved with metastatic disease.

Due to the estimated 10-year risk of recurrence of approximately 80 percent, a decision was made to treat our patient with adjuvant chemotherapy. Due to her age and the increased risk of cardiac toxicity with anthracycline-based chemotherapy regimens, our patient was treated with TC chemotherapy (docetaxel 75mg/m^2^ and cyclophosphamide 600mg/m^2^) once every three weeks for a total of four planned cycles [3].

Our patient had no other medical problems prior to her diagnosis of breast cancer aside from gastro-esophageal reflux. Her surgical history, aside from the mastectomy and lymph node dissection, included a prior appendectomy for appendicitis and complete resection of a basal cell carcinoma from her right leg. Our patient had no known prior history of pulmonary or cardiac disease. She worked as a phlebotomist, and there were no known occupational exposures to industrial chemicals that would have given her a greater risk of developing pulmonary disease.

**Figure 1:**
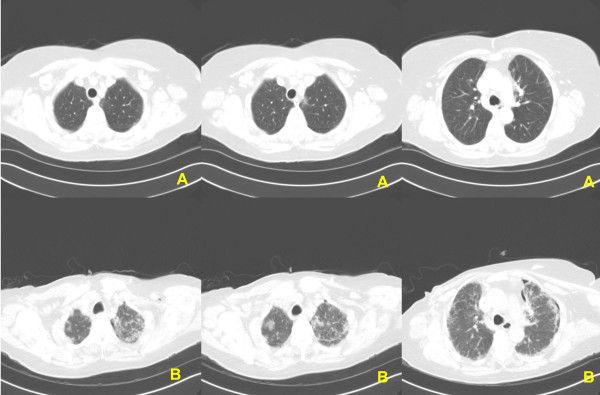
**Computed tomography scans before (A), and (B) after the third cycle of docetaxel/cyclophosphamide chemotherapy** .

Our patient had no major toxicities or issues related to chemotherapy, aside from fatigue (grade 1 to 2) and nausea and emesis (grade 2) with the first two cycles. However, approximately two weeks after receiving the third cycle of chemotherapy, our patient developed rapidly progressive dyspnea, and a non-productive cough, and went to the emergency room at an outside medical facility. She was found to have mild hypoxemia. A computed tomography (CT) angiogram was performed, which was negative for evidence of a pulmonary embolus, but did reveal the new onset of peripheral, subpleural fibrotic changes not present on pre-treatment scans (Figure 1).

**Figure 2:**
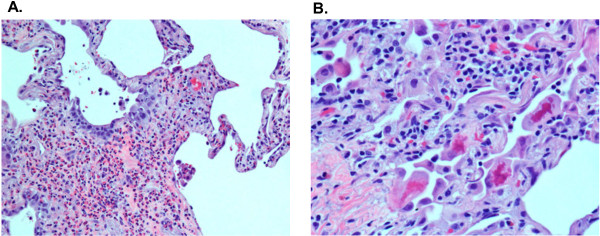
**Photomicrographs of hematoxylin and eosin stain of lung wedge biopsy. (A)** The lung interstitium is expanded by eosinophils and edema. Pneumocyte hyperplasia is observed (×200, hematoxylin and eosin stain). **(B)** Hyperplastic pneumocytes showing pink and irregular Mallory body-like inclusions (×400, hematoxylin and eosin stain).

**Figure 3:**
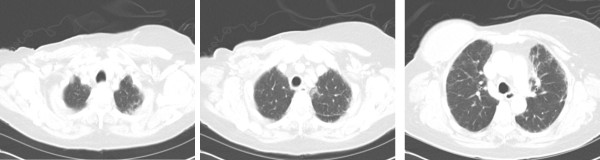
**Computed tomography scan approximately three months later showing improvement of interstitial pneumonitis** .

An attempt to pursue a needle lung biopsy at the outside hospital was complicated by a pneumothorax that required chest tube placement. Our patient was then transferred to the University of Miami hospital, for a thorascopic-guided wedge biopsy. Microscopic examination of the lung tissue revealed subacute interstitial pneumonitis evolving into areas of chronicity characterized by the presence of widening of alveolar septae with mild mononuclear infiltrates, collections of eosinophils, and pneumocyte injury (Figure 2). The latter was manifested by the presence of conspicuous Mallory body-like inclusions in hyperplastic pneumocytes. Areas of honeycombing were seen in the subpleural spaces. There was no evidence of any associated malignancy. Our patient was started on corticosteroids with rapid improvement of her dyspnea. Approximately one month later after her presentation to the emergency room, she no longer required supplemental oxygen, and had minimal dyspnea with exertion. Our patient was not given any additional chemotherapy, and was then referred for post-mastectomy radiotherapy. A CT scan performed approximately three months later showed improvement of the previously seen pulmonary infiltrates (Figure 3).

## Discussion

We present the case of a woman who developed interstitial pneumonitis (IP) after the third cycle of TC chemotherapy, with no other identifiable risk factors. Using the key words ‘docetaxel’, ‘pneumonitis’ and ‘interstitial’ on PubMed identifies 41 papers in English published in the last 15 years, of which 17 represent reports of lung toxicity in patients receiving docetaxel: 15 specifically of pneumonitis and two reports of bronchiolitis obliterans organizing pneumonia [19,20]. In those 15 papers, there were a total of 48 cases, with more than half of the cases having other lung comorbidities and no histological confirmation of the diagnosis making the diagnosis of IP uncertain (Table 1).

**Table 1:** Summary of published cases in the English literature reported as of July 2012 of docetaxel-related interstitial pneumonitis

**Reference**	**Age**	**Cancer**	**Stage IV**	**Pulmonary comorbidities**	**Chemotherapy**	**Docetaxel dose (mg/m**^**2**^**)**	**Lung biopsy**	**Steroid**	**Outcome**
[4]	44 to 77	NSCLC	Y	Y (67 percentage points)	Docetaxel	60	N	N/A	N/A
[5]	69	Prostate	Y	N	Docetaxel	75	N	Y	Death
[6]	46	Breast	N	N	TAC	75	N	Y	Resolution
[6]	64	Breast	Y	N	Docetaxel	75	N	Y	Death
[6]	52	Breast	Y	Y	Docetaxel	100	N	Y	Resolution
[6]	48	Breast	N	N	FEC then docetaxel	100	N	Y	Resolution
[7]	57	Gastric	N	Y	Docetaxel/S-1	35	N	Y	Resolution
[8]	63	Breast	N	N	DH	100	Y	Y	Death
[9]	71	GE junction	N	Y	Docetaxel	75	N	Y	Resolution
[10]	65	Breast	Y	N	Docetaxel/bevacizumab	100	Y	Y	Resolution
[11]	72	Prostate	Y	N	Docetaxel	30	N	Y	Death
[12]	U	Breast	U	U	Docetaxel	U	N	Y	Resolution
[13]	44 to 75	NSCLC	Y	Y	Docetaxel/gemcitabine	30 to 40	N	Y	Two deaths/four resolutions
[14]	78	Prostate	Y	N	Docetaxel/thalidomide	30	Y	Y	Resolution
[15]	73	Prostate	Y	N	Docetaxel	75	Y	Y	Death
[15]	74	Breast	Y	Y	Docetaxel	75	N	N	Death
[15]	61	Breast	N	N	AC then docetaxel	75	Y	Y	Resolution
[15]	54	Breast	Y	Y	Docetaxel	60	N	Y	Resolution
[16]	44	NSCLC	Y	Y	Docetaxel	33	N	Y	Death
[16]	73	NSCLC	Y	Y	Docetaxel/gemcitabine	30	Y	Y	Death
[16]	70	NSCLC	Y	Y	Docetaxel/gemcitabine	30	Y	Y	Death
[16]	75	NSCLC	Y	Y	Docetaxel	60	N	Y	Death
[18]	41	Breast	Y	Y	ThCD	125	N	Y	Resolution
[18]	48	Breast	Y	Y	ThCD	125	N	Y	Resolution
[17]	73	NSCLC	Y	N	Docetaxel	100	Y	Y	Resolution
[17]	67	NSCLC	Y	N	Docetaxel	100	Y	Y	Improvement

*AC* doxorubicin, cyclophosphamide; *DH* docetaxel, trastuzumab; *FEC* 5-fluorouracil, epirubicin, cyclophosphamide; *GE* gastroesophageal; *NSCLC* non-small cell lung cancer; *TAC* docetaxel, doxorubicin, cyclophosphamide; *ThCD* thiotepa, cyclophosphamide, docetaxel; *U* unknown.

The largest published series of docetaxel-related IP, was from Japan and reported 18 different cases of interstitial pneumonitis, out of a total of 392 patients with metastatic NSCLC treated with docetaxel (60mg/m^2^), that is, an overall incidence of 4.6 percent [4]. However, 67 percent of the patients that developed interstitial pneumonitis had prior evidence of either chronic obstructive pulmonary disease (COPD) or interstitial changes on CT scan, and the diagnosis was not confirmed by biopsy. Therefore other etiologies not related to chemotherapy, such as lymphangitic carcinomatosis, were not excluded. An exploratory analysis in this study identified a significantly increased risk of interstitial pneumonitis in patients with pre-existing radiographic evidence of emphysema (OR 4.95, P=0.016) or interstitial changes (OR 25.9, P<0.05). The authors concluded that docetaxel should not be used in patients with pre-existing interstitial changes on CT scan, however only seven of 27 of such patients developed pneumonitis, and therefore this recommendation should be viewed with some skepticism especially in patients where the overall therapeutic goal is curative and not palliative. Histopathologic descriptions due to docetaxel-related pulmonary toxicity are scant and lack details of the findings. Among the cases with histological descriptions and photomicrographs, they are limited to mentioning the non-descriptive terms of interstitial pneumonitis/fibrosis. Rare cases have shown tissue eosinophilia. Our patient showed increased numbers of eosinophils, further supporting the view of an exogenous injury such as drug toxicity as the cause of pneumonitis (Figure 2). Also, the presence of Mallory-like bodies in pneumocytes indicates that the damage at the cellular level was restricted primarily to the epithelial lining of the alveoli [22].

In our review of previously published cases of docetaxel-related interstitial pneumonitis, in most cases pneumonitis occurred after two cycles, reported as early as after the first cycle, and as late as after the ninth cycle. The overall mortality rate from drug associated interstitial fibrosis estimated from published case reports appears to be approximately 40 percent with 12 deaths in 30 cases where mortality data was available (Table 1) [15].

However, pneumonitis is a well-established toxicity of the alkylating agent cyclophosphamide [2]. The overall incidence is unknown, but has been estimated to be less than one percent of all patients who receive cyclophosphamide, with several confounding factors, including patients concomitantly receiving other drugs known to cause pneumonitis [2]. In a retrospective review from the Mayo Clinic, 35 cases in a 20-year period were found with suspected cyclophosphamide-induced interstitial pneumonitis, of which 29 were found to have other possible etiologies [21]. Of the remaining six cases, one patient had early-onset pneumonitis occurring approximately six months after initiation of cyclophosphamide, while five others developed late-onset pneumonitis, where three died and in the other two patients follow-up information was unavailable [21]. The same authors performed a retrospective review that revealed seven cases of early-onset pneumonitis and five cases of late-onset pneumonitis with a mortality rate of 29 percent in the early-onset cases, and 80 percent in the late-onset cases.

The incidence of interstitial pneumonitis in patients receiving TC chemotherapy is unknown. It is highly probable that it is a very rare complication, since thousands of patients are treated each year with both agents simultaneously, yet only a few scattered reports have emerged in the literature. Since our patient received both drugs, it is not possible to determine which agent is responsible, but the clinical, radiological and histological findings strongly support the diagnosis of drug-induced pneumonitis.

## Conclusions

TC is commonly used as a safer alternative to anthracyclines in older patients with breast cancer, because of a more favorable toxicity profile in comparison, but our patient’s case illustrates that no chemotherapy is free of risk, and that unanticipated serious complications, such as interstitial pneumonitis, can occur [3]. Fortunately, our patient made a rapid recovery and does not appear to have suffered any permanent damage. Medical oncologists should be aware that while distinctly uncommon, interstitial pneumonitis is a possible serious adverse toxicity associated with docetaxel/cyclophosphamide-based chemotherapy.

## Consent

Written informed consent was obtained from the patient for publication of this case report and any accompanying images. A copy of the written consent is available for review by the Editor-in-Chief of this journal.

## Competing interests

The authors declare that they have no competing interests.

## Authors’ contributions

RO, AM, and SG analyzed and interpreted the data from our patient regarding the development of interstitial pneumonitis and wrote the case report and discussion. PB performed the histological examination of the lung, and was a major contributor in writing the manuscript. All authors read and approved the final manuscript.
